# Enhancing autism spectrum disorder classification in children through the integration of traditional statistics and classical machine learning techniques in EEG analysis

**DOI:** 10.1038/s41598-023-49048-7

**Published:** 2023-12-08

**Authors:** Jacek Rogala, Jarosław Żygierewicz, Urszula Malinowska, Hanna Cygan, Elżbieta Stawicka, Adam Kobus, Bart Vanrumste

**Affiliations:** 1 Faculty of Physics, University of Warsaw, Pasteura 5, 02-093 Warsaw, Poland; 2 Institute of Physiology and Pathology of Hearing, Bioimaging Research Center, World Hearing Center, Warsaw, Poland; 3 Clinic of Paediatric Neurology, Institute of Mother and Child, Kasprzaka 17A, 01-211 Warsaw, Poland; 4 Institute of Computer Science, Marie Curie-Skłodowska University, Pl. M. Curie-Skłodowskiej 1, 20-031 Lublin, Poland; 5 Department of Electrical Engineering (ESAT), eMedia Research Lab/STADIUS, KU Leuven, Andreas Vesaliusstraat 13, 3000 Leuven, Belgium

**Keywords:** Autism spectrum disorders, Neural circuits

## Abstract

Autism Spectrum Disorder (ASD) is a neurodevelopmental disorder hallmarked by challenges in social communication, limited interests, and repetitive, stereotyped movements and behaviors. Numerous research efforts have indicated that individuals with ASD exhibit distinct brain connectivity patterns compared to control groups. However, these investigations, often constrained by small sample sizes, have led to inconsistent results, suggesting both heightened and diminished long-range connectivity within ASD populations. To bolster our analysis and enhance their reliability, we conducted a retrospective study using two different connectivity metrics and employed both traditional statistical methods and machine learning techniques. The concurrent use of statistical analysis and classical machine learning techniques advanced our understanding of model predictions derived from the spectral or connectivity attributes of a subject's EEG signal, while also verifying these predictions. Significantly, the utilization of machine learning methodologies empowered us to identify a unique subgroup of correctly classified children with ASD, defined by the analyzed EEG features. This improved approach is expected to contribute significantly to the existing body of knowledge on ASD and potentially guide personalized treatment strategies.

## Introduction

Autism Spectrum Disorder (ASD) is a neurodevelopmental condition distinguished by social and communication deficits coupled with restricted interests and repetitive, stereotypic movements and behaviors. These dysfunctions often result in severe difficulties in everyday functioning. ASD affects approximately 1–2% of the population, and over the past decade, there has been a steady rise in the number of diagnosed children. Autism is a heterogeneous disorder with a complex etiology involving a multitude of factors, including genetic, epigenetic, environmental, and immunological contributors. Additionally, the symptoms of ASD exhibit a high degree of heterogeneity, with varying levels of characteristic dysfunctions in individual patients.

Early detection and intervention for ASD can potentially alter developmental pathways and significantly enhance the subsequent quality of life. Furthermore, studying these preliminary biomarkers can elucidate the inherent mechanisms and plausible causative routes leading to ASD manifestations in later stages. Biomarkers serve as objective indicators reflecting standard biological processes, and they are invaluable for diagnostic purposes, prognostic assessments, and gauging the efficacy of treatments. Ensuring these biomarkers possess impeccable accuracy, consistency, and validity is imperative for their successful integration into clinical practice. Functional EEG connectivity has been suggested as a fruitful source of potential biomarkers. Functional connectivity indicates how different brain regions synchronize or communicate. It has been suggested that ASD is characterized by atypical brain connectivity from an early age. Research indicates that autistic individuals exhibit distinct brain connectivity patterns compared to control groups^1^. One prominent hypothesis suggests aberrations in functional connectivity patterns between distant brain regions in ASD subjects. Predominant literature posits the occurrence of within- and between-hemispheric long-range underconnectivity^2–4^. However, previous fMRI studies have reported inconsistent results, demonstrating evidence of both long-range hyperconnectivity and hypoconnectivity in ASD populations^5^. The unsettled debate also extends to the disturbed patterns of neuronal connections in short-range focal pathways, though contemporary research is leaning towards local hyperconnectivity in ASD. For instance, a meta-analysis of resting-state fMRI functional connectivity in pediatric ASD populations underscored consistent patterns of local under-connectivity across selected experiments, chiefly within the default mode and sensorimotor areas^6^. The outcomes highlighted in the meta-analysis imply that connectivity aberrations are functionally oriented, predominantly occurring in cortical areas responsible for cognitive and emotional processes associated with ASD symptoms. Further, task-related fMRI studies summarized in the meta-analysis by Dichter^7^ unveiled decreased activation of the "social brain" during social processing tasks, encompassing regions within the prefrontal cortex, the posterior superior temporal sulcus, the amygdala, and the fusiform gyrus in patients with ASD. Conversely, Seghatol-Eslami et al.^8^ investigated fMRI connectivity within brain networks involved in face processing, mentalizing, and mirroring. Their findings highlighted significantly greater connectivity between networks and hub regions to the rest of the brain within the ASD group. These disparities could be linked to the heterogeneous etiology of ASD and swift alterations in brain activity, which are arduous to capture using fMRI techniques. Current research consistently suggests that abnormal patterns of structural and functional connectivity are potential neural mechanisms underlying the cognitive and behavioral impairments typical in ASD patients^9–11^. Another limitation of MRI examinations is the necessity for patients to remain completely still within a narrow tube. This constraint makes MRI examinations of ASD children feasible only under sedation, which significantly alters brain activity.

EEG, an economical and widely available alternative to fMRI, offers increased availability of clinical data that can support biomarker development, given both the advantages and challenges of clinical medical testing. However, the large number of available connectivity measures yields relatively few statistically significant and meaningful correlations between each other, which argues for a re-evaluation of the methodology used in such studies^12^. In addition, the common issue of low sample sizes in clinical trials can distort statistical results, leading to inconsistent findings. While some factor-detection studies appear promising, they often fail in identifying individual cases, mainly due to methods focusing on group differences. Machine learning offers a potential solution, but requires larger trials. In order to limit the negative effects of the low concordance of different connectivity measures, small group sizes and group comparisons, our study on the classification of children with suspected ASD used features extracted from clinical EEG using two different connectivity methods: PLV^13^, taking into account both zero and non-zero phase differences, and ciPLV^14^ based only on non-zero phase correlations. And to reduce the ambiguity of classification based on small samples, we combined explainable classification methods with traditional statistical techniques. Designed for practical application in the clinic, our method was tested on retrospective data from standard clinical trials with minimal preprocessing. Concurrently applied, classical machine learning (ML) techniques facilitated predicting the likelihood of an individual belonging to the ASD group on clinical data. Furthermore, the combined use of traditional statistical methods and explained classical ML algorithms allowed for interpreting model predictions in terms of spectral or connectivity properties of a subject's EEG signal. This approach also enabled us to verify these predictions. Notably, the application of machine learning methods helped us identify a distinct subgroup of children with ASD based on the analyzed EEG features. This illustrates the potential of such techniques to study small samples not only in diagnosis but also in further understanding the heterogeneity within the ASD population.

## Materials

### Data

The retrospective study consisted of clinical EEG recordings collected from two groups of patients. The first group consisted of 18 subjects (12 boys) presenting symptoms of atypical development. EEG data were recorded between November 2017 and March 2022 at the Institute of Mother and Child in Warsaw (Poland). The average age of the children was 3.92 ± 1.38 (mean ± std), with a range of 2.08–5.92 years. Although the age range of the children was more than 3 years, the EEG correlation pattern was found to be stable at younger ages (less than 6 years) and only started to diverge at 10 years^15^. All children were diagnosed with autism spectrum disorder (ASD) and were referred to the neurology department by pediatricians or child psychiatrists for diagnostic purposes (neurological and metabolic diagnosis according to ICD 10^16^). In none of them did the parents report episodes that could correspond to epileptic seizures. In 4 patients, parents reported a sudden regression in psychomotor development, which occurred between 12 and 30 months of life. One patient was directed for suspected Pediatric Acute-onset Neuropsychiatric Syndrome (PANS), but the diagnosis was not confirmed. Fourteen children presented the most profound dysfunctions corresponding to level 3 according to the Diagnostic and Statistical Manual of Mental Disorders (DSM-V^17^) criteria (severe deficits in verbal and nonverbal social communication skills, very limited initiation of social interactions, minimal response to social taunts from others, and marked anxiety when rituals or routines are interrupted). In the remaining six children, clinical assessment identified deficits in social interaction communication and difficulties in verbal and non-verbal communication, even with support (level 2). In 12 children, cognitive development was scored as being within the standard range (in 9 according to the Leiter scale^18^, in 3 based on clinical examination and observation), in 2 as borderline age-standard, and one child was diagnosed with mild intellectual disability. Collected EEG was assessed by clinical neurologists, and no symptoms of abnormal EEG characteristics were identified.

The second group consisted of children whose recordings were selected from the EEG database of Warsaw University. Clinical recordings were collected between November 2011 and August 2018 in different hospitals from 18 children (12 boys) with an average age of 3.84 ± 1.24 (mean ± std), range: 2.06–4.92 years. All children in the second group were identified as typically developing (TD) with no symptoms of abnormal EEG characteristics. Children for the TD group were selected to match the age, sex, and sleep stages observed during the EEG recording of the children from the group presenting symptoms of atypical development.

None of the clinical descriptions associated with analyzed EEG recordings provided information on the duration or percentage of time attributed to the different stages of sleep observed by the clinicians. This was the case for both typically developing children and children with autism. Therefore, we decided to lump them together for further analysis. The groups did not differ in terms of age (Wilcoxon Z = − 0.36, *p* = 0.7) or gender distribution (two sample Smirnov-Kolmogorov test STAT = 0.5, *p* = 1).

The local bioethics committee at the Mother and Child Institute in Warsaw approved the study and use of retrospective data. The Bioethics Committee has granted an informed consent waiver at the Institute of Mother and Child in Warsaw by Opinion No. 3/2023 dated 26.01.2023. The electronic copy of the original decision has been attached to the manuscript. All data were anonymized, and names and other HIPAA identifiers were removed from all manuscript sections, including supplementary information. Manipulation of retrospective data was performed in accordance with all relevant guidelines and regulations.

### Preprocessing

All EEGs were recorded with a sampling rate of 250 Hz and 19 electrodes in a 10–20 system; according to the routine medical procedure the impedance of the electrodes should not exceed 5 KΩ. Preprocessing included 1–45 Hz filtering (Hamming windowed FIR filter with heuristic order/transition bandwidth estimation implemented in EEGlab) and exclusion of data segments containing large muscle and movement artifacts detected by visual inspection. Filter order and transition bandwidth were estimated using heuristic, including transition bandwidth of 25% of the lower passband edge, but not lower than 2 Hz, where possible (for bandpass, highpass, and bandstop) and distance from passband edge to critical frequency (DC, Nyquist) otherwise. Bad electrodes were interpolated using EEGlab spherical interpolation function (pop_interp^19^). To account for various placements of the reference electrodes, all recordings were re-referenced to the Fz electrode, yielding 18 EEG channels for the analyses. We did not apply the average reference method since it mixes signal phases and, therefore, is not recommended for any phase correlation measures. For the analyses, only eyes closed epochs were extracted from the recording based on the markers inserted manually by clinicians during the session. The epochs were then divided into 2-s-long segments to account for lower band frequencies while assuring maximum signal stationarity.

## Methods

### Spectral analysis

Spectral analyses included a comparison of average power from 2-s windows, all channels, in the standard frequency bands: theta (θ; 4–7 Hz); alpha (α; 8–12 Hz); low beta (β1; 13–20 Hz); high beta (β2; 21–30 Hz). All power estimates were computed using the pwelch method (with a Hamming window) and normalized (rescaled range of data to 0–1) in each 2-s segment across all electrodes. The obtained spectra were averaged across all segments for a given subject.

Friedman test for groups of subjects and channels was performed separately for each frequency band. The mass-univariate test, a 2-tailed Wilcoxon rank sum test with the false discovery rate (FDR) correction^20^, with the threshold of p = 0.05, was performed as a post-hoc in search for channels and frequency bands with a significant difference in power between groups.

### Measures of functional connectivity

Functional connectivity was estimated in the same EEG bands as the spectral features. As a measure of functional connectivity, we used phase-locking value (PLV^13^) commonly used to estimate connectivity in EEG/MEG studies^21–24^. PLV shows robustness to noisy signals^25^ commonly present in clinical practice; its results were also shown to relate significantly to the results of fMRI connectivity analyses^26^. To compute PLV in a chosen frequency range, we filtered the EEG data using pass-band FIR filters with an order adjusted for each frequency band (theta, alpha, and low beta filter order = 24, high beta filter order = 50) and then obtained the analytical signal using Hilbert transform. The instantaneous phase of the channel $i$, ${\phi }_{i}(t)$ , was computed as the complex argument of the analytical signal, and it was used for PLV computation according to the formula:

$${PLV}_{ij}=\frac{1}{T}\left|\sum_{t=1}^{T}{e}^{-i\left({\phi }_{i}\left(t\right)-{\phi }_{j}\left(t\right)\right)}\right|$$

where $T$ is the data length. PLV was evaluated for 2-s epochs and averaged across all segments for a given subject, yielding a 153 feature vector for each frequency band (153 = ½(18 × 17) is the number of unique pairs of channels).

The standard PLV measure is known to be influenced by volume conduction, which has zero phase difference; therefore, to extract the information related only to non-zero-lag connectivity, we also evaluated the measure ciPLV proposed by Bruña et al.^14^:

$${ciPLV}_{ij}=\frac{\frac{1}{T}Im(\sum_{t=1}^{T}{e}^{-i({\phi }_{i}\left(t\right)-{\phi }_{j}(t))})}{\sqrt{1-{\left(\frac{1}{T}Re\left(\sum_{t=1}^{T}{e}^{-i({\phi }_{i}\left(t\right)-{\phi }_{j}(t))}\right)\right)}^{2}}}$$

where $Re(.)$ and $Im(.)$ are the real and imaginary parts of the argument. The statistical inference was done with massive univariate tests. All comparisons were performed using a 2-tailed Wilcoxon rank-sum test corrected for multiple comparisons using the FDR method with the threshold of p = 0.05.

### Classification methods

We performed tests of applicability of the spectral and connectivity measures as features for classifying subjects into the ASD or TD group of children. We utilized a simple logistic regression model with L2 regularization for each feature type. The model estimation and assessment were performed with nested cross-validation with 6 outer and 5 inner splits. The inner loop optimized the model parameters and its regularization parameter using a grid search. The outer loop was used to get the estimates of the optimized models’ performance in terms of accuracy (ACC), specificity, sensitivity, and area-under-the-curve (AUC) metrics.

The regularization is necessary to prevent the algorithm from overfitting the training dataset since the models are trained on more numerous features than the number of available examples in the datasets. In the case of spectral features, the input vector was of size 72 (= 18 electrodes × 4 frequency bands). In the case of the PLV and ciPLV measures, it was of size 612 (= 153 pairs of electrodes × 4 frequency bands). Further, we will refer to the models as spectral or PLV classifiers, respectively. We explained the models using the Shap^27,28^ technique. Since the features utilized by the models are correlated, we applied the full conditional SHAP values (SV) that respect the correlations among the input features, so if the model depends on one input, but that input is correlated with another input, then both get some credit for the model’s behavior. To aggregate the SV across subjects and obtain the group-wise assessment of features utilized by the models, we evaluated the feature importance (FI). FI was obtained as a mean of absolute values of SV across the subjects and then normalized by dividing by maximal FI. FI was analyzed separately for spectral, PLV, and ciPLV features.

Finally, based on the leave-one-out technique (LOO), we obtained the prediction for each subject resulting from the model trained on data from all the other subjects.

## Results

### Comparison of EEG spectral components

**Figure 1:**
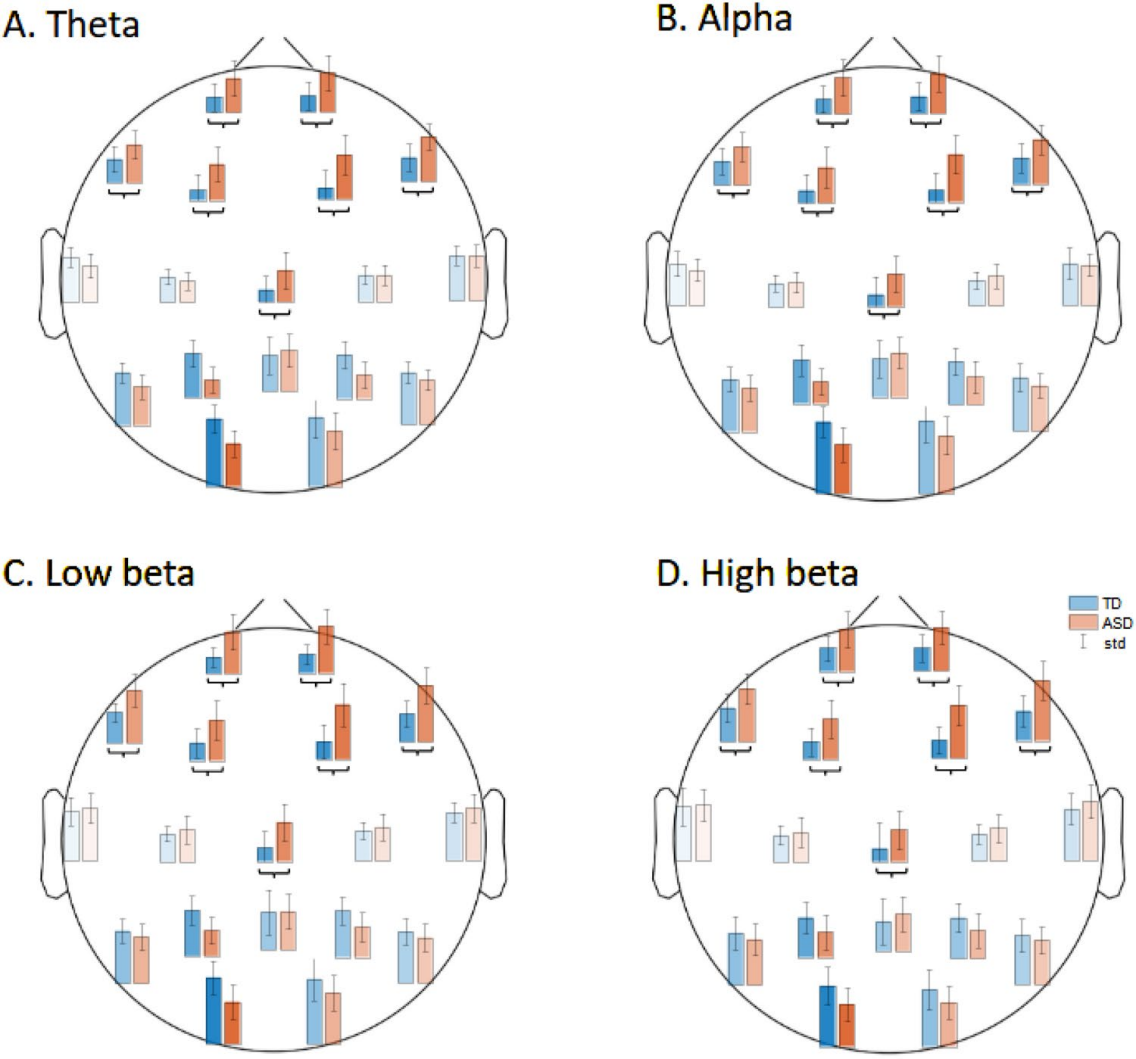
Distribution of the median power ± std in (**a**) theta, (**b**) alpha, (**c**) low beta, and (**d**) high beta bands across all electrodes in groups of children with ASD in red and TD ones in blue. Black brackets mark channels with significant differences between groups showing higher average power in the ASD group. The opacity of the bars is proportional to the FI of the spectral power at a given electrode and frequency band.

Friedman tests with factors: channels (18 levels) and groups (2 levels TD, ASD) were performed for each frequency band separately and showed significant effect of channels and groups on theta (χ^2^(17) = 163.99, *p* < 0.001), alpha (χ^2^(17) = 156.69, *p* < 0.001), low beta χ^2^((17) = 131.4, *p* < 0.001) and high beta(χ^2^(17) = 129.62, *p* < 0.001) power. The difference between groups was also found for all frequencies: theta (χ^2^ (1) = 5.69, *p* = 0.017), alpha (χ^2^(1) = 14.58, *p* < 0.001), low beta (χ^2^ (1) = 21.45, *p* < 0.001), and high beta (χ^2^(1) = 24,96, *p* < 0.001). The mass univariate tests (Wilcoxon rank sum test with FDR correction) performed as post-hocs showed that in Fp1, Fp2, F7, F3, F4, F8, and Cz electrodes, the power for all frequency bands was higher for ASD than for TD individuals (Fig. 1). What is interesting, while in TD subjects we observed an increasing trend in power from frontal to posterior electrodes, for ASD ones the level of activity in those frequency bands remains similar across electrodes.

### Comparison of connection strengths

Group comparison between TD and ASD children yielded significant differences in all analyzed EEG bands using both measures (PLV and ciPLV).

**Figure 2:**
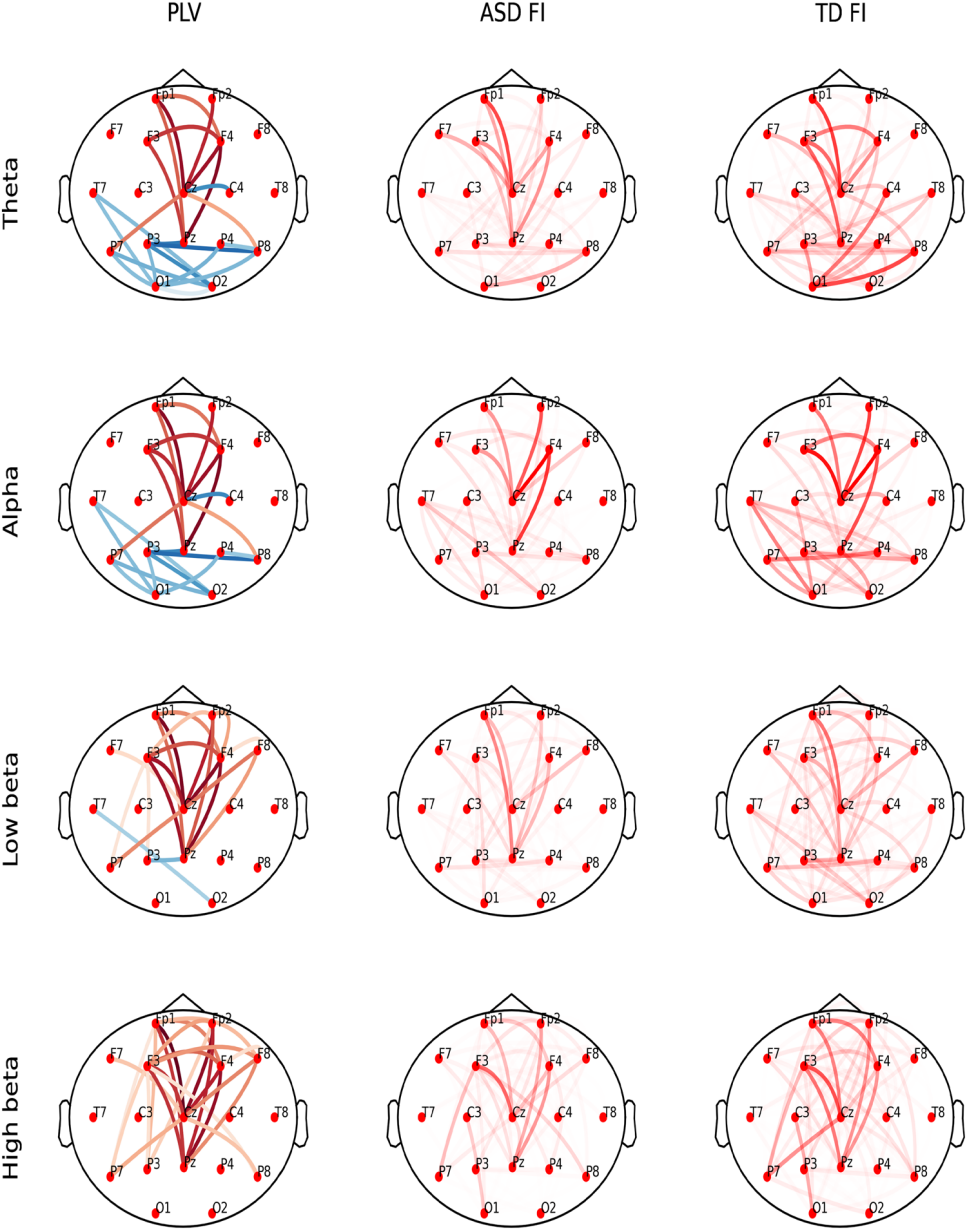
Patterns of important PLV. Left column: Significant differences in connectivity estimated by PLV between ASD and TD children. The red color denotes a larger PLV for a given pair of electrodes in ASD than in TD children, and the blue color denotes a smaller PLV in ASD than in TD children for a given pair of electrodes; the intensity of the color is proportional to the value of the difference. All marked differences are significant at *p* < 0.05, FDR corrected. Middle column: FI for ASD group; right column: FI for TD children; opacity is proportional to FI^4^ to increase the contrast.

PLV analyses showed connectivity between frontal and central parietal areas with two main hubs at the Cz and Pz electrodes. Most connections showed higher PLV in ASD children. The number of significant connections increased with the frequency of analyzed EEG bands (Fig. 2, left column).

**Figure 3:**
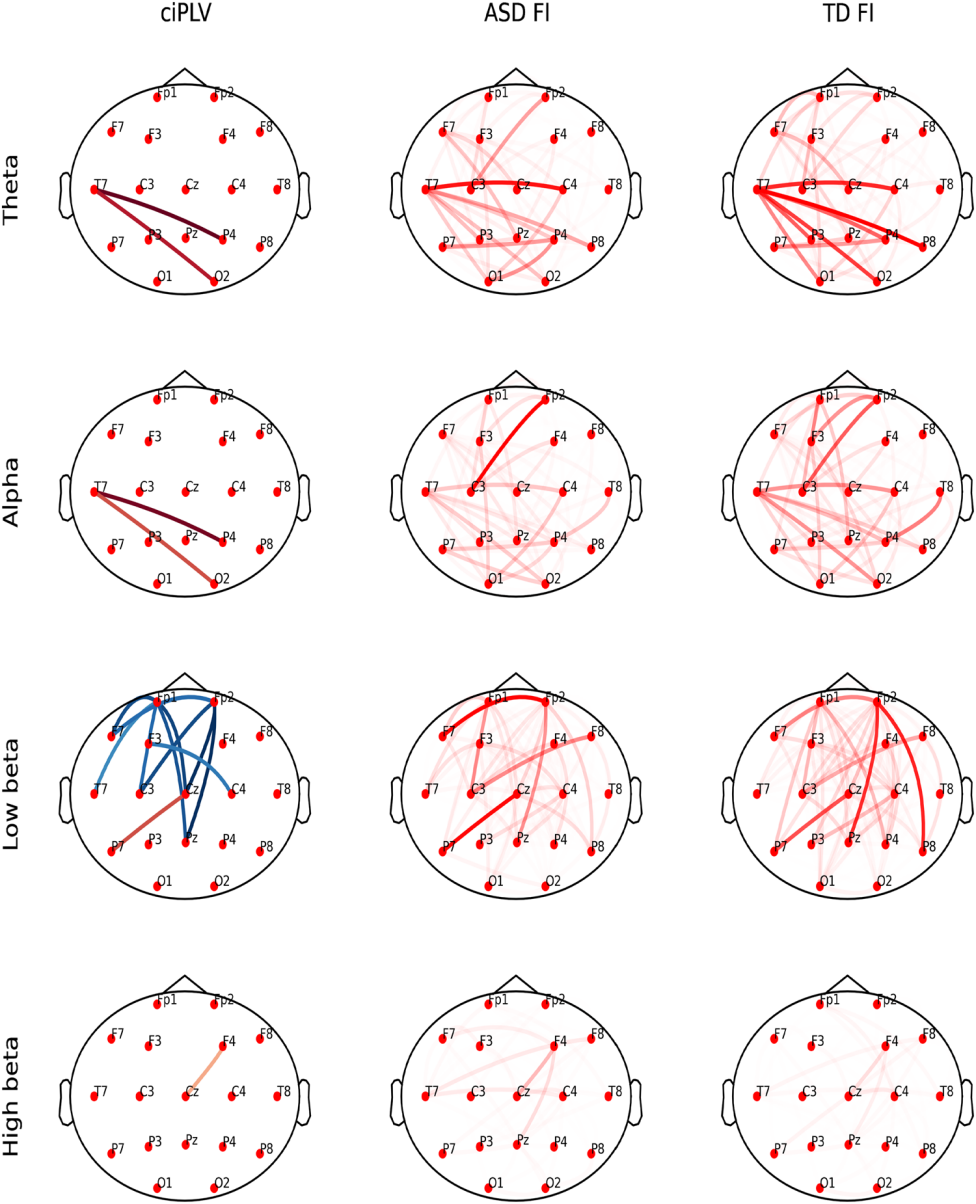
Patterns of important ciPLV. Left column: Significant differences in connectivity estimated by ciPLV between ASD and TD children. The red color denotes a larger ciPLV for a given pair of electrodes in ASD than in TD children; the blue color denotes a smaller ciPLV in ASD than in TD children for a given pair of electrodes; the intensity of the color is proportional to the value of the difference. All marked differences are significant at *p* < 0.05, FDR corrected. Middle column: FI for ASD group; right column: FI for TD children; opacity is proportional to FI^4^ to increase the contrast.

ciPLV analyses showed the most pronounced group differences in the low beta band. Unlike the PLV method, most connections showed lower strength in ASD children (Fig. 3, left column).

### Classification results

#### Metrics of classification quality

Cross-validation analyses yielded similar results for all types of input features. Detailed classification results obtained in a nested d cross-validation for spectral and both connectivity measures are presented in Table 1.

**Table 1:** Metrics of ASD developing children classification quality in nested cross-validation for spectral PLV and ciPLV features [mean (std)].

	Spectral	PLV	ciPLV
Accuracy	0.83 (0.14)	0.83 (0.17)	0.86 (0.18)
Sensitivity	0.83 (0.17)	0.78 (0.17)	0.89 (0.16)
Specificity	0.83 (0.17)	0.89 (0.25)	0.83 (0.25)
AUC	0.83 (0.14)	0.83 (0.17)	0.86 (0.18)

#### Explanations of the models

To validate the results, we analyzed FI indexes to ensure that the models pay attention to features identified in the classical statistical analysis. In the case of spectral features, we used the FI as opacity for the bars presenting the differences of median power in Fig. 1. We can observe that all statistically significant differences also have relatively high FI, but additionally, the model utilized information from P7, P3, P4, and O1 electrodes, especially in theta, alpha, and low beta frequency bands. We may note that in these additional channels, the median power also shows differences between the ASD and TD children, but those differences did not reach statistical significance.

In the case of PLV and ciPLV features, we contrasted the obtained FI for ASD and TD children with the statistically significant differences between the values of those two groups in Figs. 2 and 3. Again; we can observe the qualitative similarity of patterns of connections visible in corresponding subplots for a given frequency band.

#### Predictions for individuals

**Figure 4:**
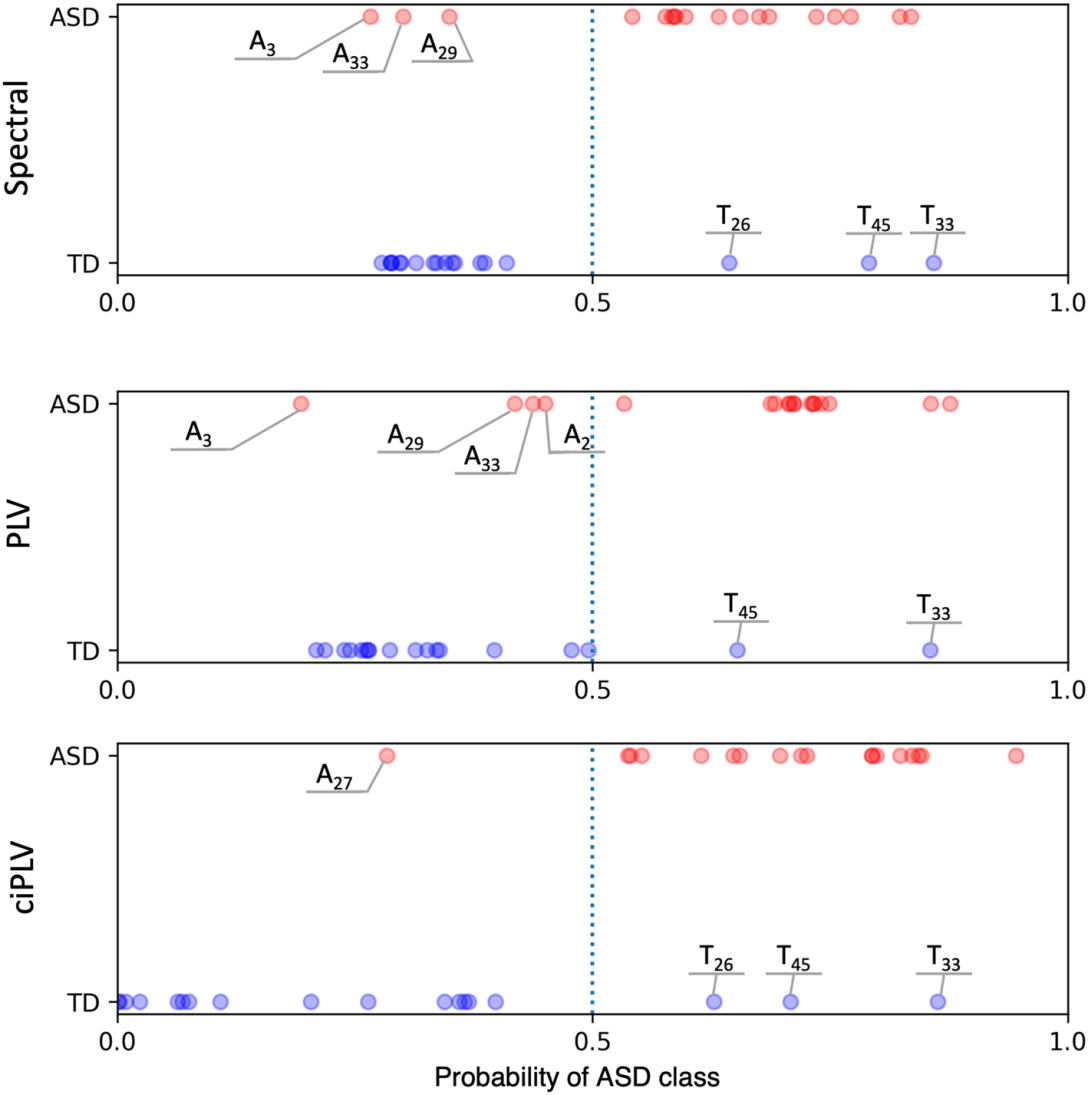
Probability of class ASD (horizontal axis) in LOO for models utilizing spectral, PLV, and ciPLV features. Blue dots mark individuals from TD and red from ASD groups; dotted vertical lines mark the probability equal to 0.5. Misclassified individuals are tagged with their labels.

**Figure 5:**
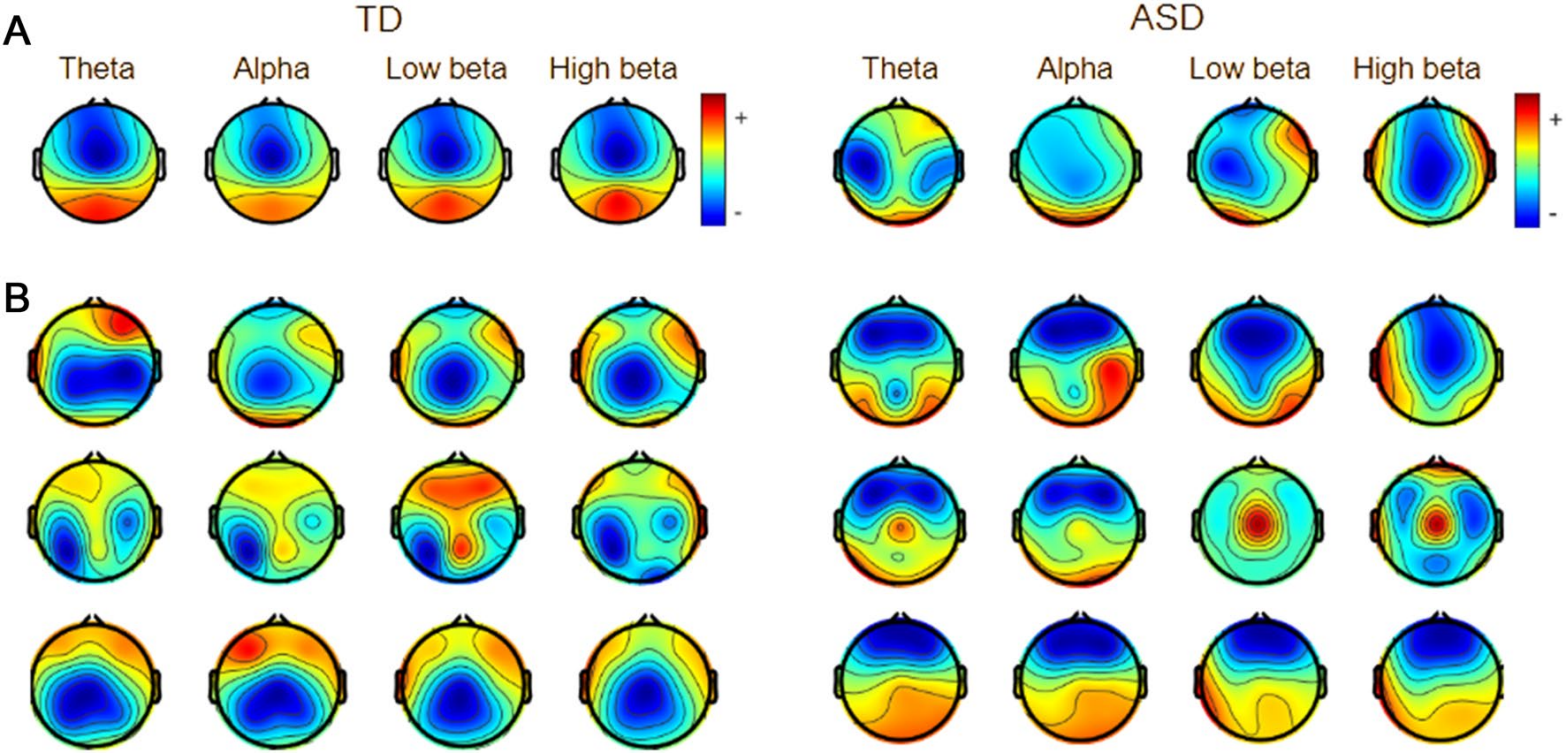
Spectra maps for subjects in groups of TD children on the left and ASD children on the right. (**a**) examples of the spectra map for one of the best-classified subjects from the two groups, (**b**) three subjects from each group who appeared as misclassifications.

The relatively high values of metrics of classification quality and plausible explanations of the models encouraged us to investigate the predictive power of the models for individual cases. To this end, we performed LOO analysis, predicting results for individual subjects using models trained on all other subjects. The results of the LOO analysis are presented in Fig. 4. The horizontal axis indicates the probability of a given case being in the ASD class. It can be observed that there is a clear grouping of subjects from the ASD group in the higher (above 0.5) range, while the subjects from the TD group cluster in the lower (below 0.5) range of the horizontal axis. The misclassified cases are mostly the same for all classifiers. As presented in Fig. 5, the misclassified subjects in both groups present a deviating pattern of spectral maps from those characterized by the best-classified subjects in the groups and correspond more to the opposing group.

## Discussion

Early detection and intervention for Autism Spectrum Disorder can potentially alter developmental pathways and enhance the subsequent quality of life. Consequently, pinpointing infantile biomarkers capable of forecasting an autism diagnosis or indicating dimensional variations in pertinent traits during subsequent development is highly important. Biomarkers serve as objective indicators reflecting standard biological processes, and they are invaluable for diagnostic purposes, prognostic assessments, and gauging the efficacy of treatments. Ensuring these biomarkers possess impeccable accuracy, consistency, and validity is imperative for their successful integration into clinical practice. To achieve this goal, studies are performed in highly controlled laboratory environments using carefully selected samples and many experimental paradigms and methods. However, in clinical practice, the application of laboratory methods is very limited.

On the other hand, the use of clinical data for investigations is hampered by their limited availability and rigid medical paradigm. This condition invariably impairs their statistical power, diminishing the reliability of any inferences drawn. Traditional statistical methods, frequently employed in these investigations, bear an additional limitation: group-based inference. Despite the possibility of producing highly reliable results, these methods may overlook individual variances that contribute to the full spectrum of a disorder. On the positive side, traditional statistical methods allow for precisely identifying key features, fostering a deeper comprehension of the underlying mechanisms at work. In contrast, machine learning techniques, which could be considered a counterpart to traditional statistical methods, facilitate individual case classifications. However, they typically demand large sample sizes, and interpreting the importance of features behind these classification outcomes can be challenging.

To minimize the above risks, we combined traditional statistical methods and explainable machine learning (ML) methods and performed cross-validation of features obtained by both methods. Our search for early indicators of ASD was based on EEG connectivity in resting-state conditions using clinical data. We selected EEG connectivity for the classification and statistical and ML inferences as the connectivity differences between typically developing children and those with ASD have been identified as one of the most commonly detected using traditional statistics^12,15,29–36^. Although EEG connectivity delivers promising results, due to a lack of correlations between different methods^12^, studies using different methods have produced somewhat inconsistent results regarding the nature of these differences or even show a lack of associations between EEG connectivity and ASD^37^. Another possible reason for disparities between different studies using connectivity metrics is the paradigm difference. Many investigations used active paradigms where children were exposed to stimuli while EEG signal was recorded^32,33,37–41^, while others used resting-state or sleep data for their analysis^30,31,34,42^.

To account for connectivity metrics inconsistencies, we applied two different methods: PLV^13^, considering both zero and non-zero phase correlations, and ciPLV^14^, based only on non-zero phase correlations. In addition, we also used spectral power features to check for their relevance for classification. Employed in our study, classical machine learning algorithms, which are more suited for smaller datasets^43,44^, yielded consistent classification results (Tab. 1). Furthermore, comparison of misclassified individuals also showed high consistency, indicating the relevance of features selected for classification. However, our results turned out to be lower as compared to other classification studies. For instance, Alotaibi and Maharatna^45^, using PLV and support vector machine (SVM), obtained an accuracy of 95.8% on the group with 12 autistic and 12 TD children aged 6–13. However, besides the age difference, Alotaibi and Maharatna^45^ used task data and high-density EEG with 128 electrodes in laboratory conditions, which could favor better classification scores. Also, other studies using different ML methods and datasets scored relatively higher than our results. For instance, Haputhanthri et al.^46^, in a group of 15 participants (10 with ASD) between 5 and 17 years of age, obtained 93% accuracy using random forest on discrete wavelet transforms from task data. One of the few studies using larger groups (46 autistic and 63 TD) with the age of children (3–5) matching our study was the work of Zhao et al.^47^. The authors used alpha band singular spectrum analysis of laboratory-collected and preprocessed data using SVM. The ASD versus TD classification accuracy was 92.7%.

Although the classification results of all of the studies mentioned above are higher than ours, they all used data cleaned from artifacts and collected in the laboratory during either task or eyes-open conditions, which is not feasible in clinical practice.

Our statistical counterpart analysis based on the same metrics yielded significant differentiating connectivity patterns. PLV analyses showed increased connectivity on all significant connections in children with ASD as compared to the TD control group—contradictory to previous studies investigating the neural correlates of ASD. These disparities could stem from many factors, one of them being the sleep data used for analyses in our study, while many studies showing decreased connectivity in children with ASD used task data^15,33,39,41^. Other possible reasons for these disparities may include low sample size, where a few subjects skew the results, high group heterogeneity, a wide range of methodologies employed in experiments examining resting-state EEG abnormalities in ASD^9^, or a combination of all these factors. Interestingly, the same analysis with the debiased ciPLV connectivity method also allowed us to differentiate the TD and autistic children. However, the connectivity pattern showed decreased values in autistic children in the low beta band.

To clarify the situation and either confirm or refute our findings, we employed explainable ML methods to extract features important for classification and compared them to the results of statistical analyses. Notably, we observed high consistency across all effects concerning power spectra and PLV analysis. Also, comparing features extracted from ciPLV classification and its statistical counterpart showed qualitative similarities. Although this consistent alignment between the importance of features and statistical analyses is encouraging, the classifier identified a small subgroup of children within the ASD group with a low probability of ASD. This may be explained by several factors, one of which could be the group's heterogeneity, characterized by different EEG features associated with various neuro-subtypes^48^.

However, a comparison of misclassified individuals between PLV and ciPLV methods showed high consistency in misclassified TDs, which might suggest the validity of both PLV and ciPLV approaches. Although the most serious objection to the PLV method is the confound of volume conduction, our results and studies showing that zero-phase correlations present in the PLV provide information enabling prediction of the task results, not possible otherwise^49^ and that PLV and other zero-phase correlations methods are significantly related to the results of fMRI analyses^26^ indicate their physiological relevance allowing for classification results matching those of debiased connectivity metrics.

A comparison with recent EEG-based classification studies shows how diverse the groups compared are and how different methods in terms of classifiers and features are used. Among the studies published in recent years (Table 2), most were based on groups with a wide age range or unbalanced in terms of participants with TD and ASD. All studies were conducted under resting-state in laboratory conditions to ensure the least possible contamination by artefacts. None of these studies used techniques to interpret the classification results to ensure that the classification was not based on artefacts^50^. We found only one study^47^ that involved children diagnosed for ASD using similar methods and at a similar age as in our study. The accuracy obtained by the authors, on artefact-cleaned signals recorded in the resting-state, ranged from 81 to 92%, depending on the features used. As the authors did not perform the LOO procedure, it is difficult to determine whether the classification yielded similar participants for the different features or whether routine statistical analysis would have shown significant differences between groups on the basis of the studied features. Our study was also the only one to use raw signals collected from sleeping children during routine clinical procedures.

**Table 2:** Comparison of ASD classification studies using EEG.

Source	Age range	Group size	ASD diagnosis method	Classifier	Accuracy
Current study	ASD /TD: 2–6	ASD: 12 boys, 6 girls. TD: 12 boys, 6 girls	DSM-V, PANS, evaluation of clinical professionals, medical history	LR with L2 regularization	83–86
Zhao et al.^47^	ASD /TD: 3–5	ASD: 23 boys, 23 girls; TD: 33 boys, 30 girls	DSM-V, CARS	SVM	81–92
Ari et al.^51^	ASD: 6–20, TD: 9–13	ASD: 20. TD: 9	Not available	ResNet101	98.32
				ResNet50	97.20
				ResNet18	98.88
Peya et al.^52^	ASD/TD: 7–12	ASD: 13 boys, 2 girls. TD: 4 boys 6 girls	DSM-V	ResNet	100
Baygin et al.^53^	ASD/TD: 4–13	ASD: 61; TD: 61	evaluation of clinical professionals, medical history, international scales	SVM	96.44
Tawhid et al.^54^	ASD: 6–20, TD: 9–13	ASD: 3 girls, 9 boys. TD: boys 4	Not available	NB	72.09
				LDA	89.97
				RF	90.59
				kNN	92.29
				LR	94.95
				SVM	95.25
				CNN model 1	96.16
				CNN model 2	96.02
				CNN model 3	99.15

SVM: Support Vector Machine, ResNe: Residual Neural Network, NB: naïve bayes, LDA: linear discriminant analysis, RF: random forest, kNN: k-nearest neighbor, LR: logistic regression, CNN: convolutional neural network.

In summary, our analysis of spectral features and two different connectivity indices using a logistic regression model interpreted using the Shapley technique^27,28^ revealed unique and consistent subgroups of connectivity methods among children identified as having ASD. These subgroups showed a high probability of ASD, and their patterns of EEG activity were consistent with those identified in our traditional analyses. Conversely, the second subgroup was classified with less certainty, as they showed EEG features that more closely resembled those found in the TD control group. The high consistency of correctly classified and misclassified TD subjects demonstrates the physiological relevance of the two connectivity metrics and the feasibility of using combined machine learning and statistical methods to infer from small data samples.

## Conclusions

Analyzing small datasets using a variety of metrics is a common challenge in clinical and research practice and inherently carries the risk of producing inconsistent, unstable, and poorly generalizable results. To reduce uncertainty in inference, it may be crucial to integrate traditional statistical methods and classical machine learning techniques further enhanced by explanation methods. Combining different methodologies can generate robust and reliable inferences, increasing confidence in the results. The consistency of the differentiation of features by both methods not only strengthens the reliability of the analysis but also promotes a better understanding of the available data. Furthermore, the ability to classify individual cases is a significant advantage. It also helps to stratify groups, which is necessary for spectrum disorders. Therefore, this multifaceted approach not only reduces risk but also adds value, leading to a deeper, more nuanced understanding of the data.

### Limitations

The autism diagnosis of children referred to as ASD was based on ICD 10 and may not be very specific; therefore, some children might have another disorder than ASD.

Other limitations of this study include (i) the lack of precise data on sleep onset, brakes, or staging, (ii) the risk of overfitting associated with a small sample size and much larger set of features, (iii) the study includes recordings collected in different hospitals which may introduce other confounding factors not accounted for in our analyses.

## Data Availability

The datasets used and analyzed during the current study are available from the corresponding author upon reasonable request.
